# Supporting a review of the benefits package of the National Health Insurance Scheme in Ghana

**DOI:** 10.1186/s12962-022-00365-0

**Published:** 2022-07-16

**Authors:** Heleen Vellekoop, Emmanuel Odame, Jessica Ochalek

**Affiliations:** 1 Institute for Medical Technology Assessment, Erasmus University Rotterdam, P.O. Box 1738, 3000 DR Rotterdam, The Netherlands; 2 Ministry of Health, Accra, Ghana; 3 Centre for Health Economics, University of York, York, UK

**Keywords:** Health benefits package, Financial risk protection, Political economy, LMIC, Ghana

## Abstract

**Background:**

Although Ghana is lauded for its National Health Insurance Scheme (NHIS), concerns exist about the scheme’s functioning and sustainability. An often-cited issue—contributing to the scheme’s decreasing membership, long-standing financial deficit, and frequent out-of-pocket payments among members—is the large benefits package (BP). While, on paper, the BP covers over 95% of the conditions occurring in Ghana, its design was not informed by any budget analysis, nor any systematic prioritization of interventions. This paper aims to provide evidence-based input into ongoing discussions regarding a review of the NHIS benefits package.

**Methods:**

An existing analytic framework is used to calculate net health benefit (NHB) for a range of interventions in order to assess their cost-effectiveness and enable the prioritization of ‘best buys’. The framework is expanded upon by incorporating concerns for financial protection, and practical feasibility, as well as the political economy challenges of disinvesting in currently funded activities. Five different options for the benefits package, each based on policy discourse in Ghana’s health sector, are presented and evaluated.

**Results:**

Implementing all interventions for which data was available to 100% of the population in need was estimated to cost GH₵4323 million (US$994 million), while the available NHIS budget was only GH₵970 million (US$223 million). Options for the benefits package that focussed on cost-effectiveness and primary care provision achieved the best health outcomes, while options reflecting the status quo and allowing for co-payments included a higher number of healthcare interventions. Apart from the package option focussing on primary care, all packages were faced with physician shortages.

**Conclusions:**

Current funding to the NHIS is insufficient to provide the historical benefits package, which promises to cover over 95% of disease conditions occurring in Ghana, to the total population. Shifting the NHIS focus from intervention coverage to population coverage is likely to lead to better health outcomes. A primary care package may be most feasible in the short-term, though additional physicians should be trained to provide higher-level care that is highly cost-effective, such as emergency neonatal care.

**Supplementary Information:**

The online version contains supplementary material available at 10.1186/s12962-022-00365-0.

## Background

In 2004, Ghana rolled out its National Health Insurance Scheme (NHIS) to replace the prior ‘cash-and-carry’ system, in which healthcare costs were mostly covered through out-of-pocket payments (OOPs). The NHIS was designed with voluntary enrolment and no user fees at the point of healthcare delivery. While studies have found increased healthcare utilization and a higher level of financial protection among the enrolled compared to the non-enrolled population [1–7], concerns exist regarding the performance and sustainability of the NHIS.

For example, NHIS enrolees are supposed to receive healthcare for free at the point of delivery, yet frequent (catastrophic) OOPs are reported [7, 8]. Health facilities charge out-of-pocket fees partly because claims reimbursement to the facilities is unpredictable and generally several months late. The National Health Insurance Authority (NHIA), which administers the NHIS, has been running financial deficits since 2010 [9]. Among the reasons identified for the financial issues is the large benefits package [9, 10]. According to the NHIA, the current benefits package covers over 95% of disease conditions occurring in Ghana [11]. However, the design of the existing benefits package was based on limited technical analysis [12]: no financial analysis was done to align the size of the benefits package with the available budget, nor were methods of health technology assessment (HTA) used to decide on the in- and exclusions for the package.

Out of the three dimensions of healthcare coverage that the World Health Organization identified [13], Ghana’s NHIS—with its large benefits package and pledge of no user fees—has historically concentrated on *service coverage* and *cost coverage*. *Population coverage* appears to have stagnated and hovered around 40% from 2015 to 2020 [9, 10]. Nonetheless, Ghana has been focusing on strengthening its primary healthcare system in recent years, in a bid to make a basic level of health services available to all citizens [14].

Considering the aim of expanding population coverage and given the financial woes of the NHIS, policymakers have been discussing a review of the NHIS benefits package. Over recent years, two main ideas in policy-making circles have been: a benefits package consisting only of primary care interventions, and; a tiered system, in which a basic package is provided for free and an additional package comes with co-payments. Moreover, increased efficiency in the health sector is frequently mentioned as a policy aim [14]. In this paper, the analytic framework developed by Ochalek et al. [15] is applied to provide evidence-based input into discussions regarding a review of the NHIS benefits package. Ochalek et al. recommend prioritizing healthcare interventions according to the net health benefit (NHB) they generate, a measure of population health. The framework is expanded upon in this study, by also including outcome measures reflecting concerns for financial protection, practical feasibility, and the political economy challenges of disinvesting in currently funded activities. Five different options for the benefits package are presented, each based on recent discourse in Ghana’s health sector.

## Methods

The study methodology consisted of four main steps:

1. A list of healthcare interventions was compiled. Data was gathered on the health gains, costs, and population in need for each intervention. Subsequently, the budget impact when providing the intervention to the full population in need was calculated, as well as the NHB and the number of physicians required.
2. Five different benefits packages were assembled. Each of the packages focused on different goals and so included a different set of healthcare interventions.
3. Outcomes were calculated for each benefits package scenario, using four different measures. Two measures reflected nation-wide outcomes: ‘annual total NHB’ and ‘net physician shortage’. The other measures described benefits package-specific outcomes: ‘number of interventions covered by the benefits package’ and ‘annual number of cases treated through the NHIS’.
4. The sensitivity of the outcomes for the package scenarios to changes in key input parameters was assessed using one-way sensitivity analysis (SA).

### Step 1: outcomes at the intervention level

Table 1 shows the data sources used in estimating outcomes for the included healthcare interventions. A list of interventions to be considered for inclusion in the benefits package was based on the OneHealth Costing Tool. A literature search was conducted in the Global Health CEA Registry to gather data on the health outcomes for each intervention. Incremental health outcomes were calculated by comparing the intervention to a situation of ‘no healthcare’, as the benefits packages in this study are assumed to be designed de novo (i.e. from scratch) [16]. The disability-adjusted life-year (DALY) was adopted as the metric reflecting health, as DALYs are most used in low-income countries. Interventions for which no studies using the DALY metric were available, or for which studies provided insufficient information to be able to adapt the findings to the Ghanaian setting, were excluded from consideration for the benefits package (see Additional file 1: S1 for details).

**Table 1:** Data sources used in estimating health and cost outcomes per intervention

Data item	Source	Comments
List of healthcare interventions	OneHealth Costing Tool v4.53 [22]	
Health outcomes (DALYs avoided) per healthcare intervention	Academic literature (cost-effectiveness studies)	Full list of references available in Additional file 1: S2
Costs		
*Medicines*	NHIS Medicines List, and wholesale price quotes	– The most recent NHIS Medicines List can be accessed through: https://www.nhis.gov.gh/medlist.aspx – Costs for relevant pharmaceuticals not covered by the NHIS were provided by three wholesale providers of pharmaceuticals through a standardized Excel file
*Consumables*	Procurement departments of health facilities, and wholesale price quotes	Costs were provided by two health facilities and five wholesale providers of medical goods through a standardized Excel file
*Lodging during in-patient stay*	Ridge Hospital in Accra, Ghana	
*Health worker wages*	Payroll data from the Ministry of Health	Based on the 2017 period, average monthly salaries were calculated for each type of health worker and multiplied by 12 to obtain yearly health worker wages
Resource use per case treated	OneHealth Costing Tool v4.53 [22], validated by experts	Three Ghanaian health professionals provided feedback on an initial list of resource use per case treated for each intervention that was based on data in the OneHealth Tool: – A medical doctor gave input on the specific activities within each intervention (e.g. number of consultations), as well as the type of health worker providing the activities, the time spent on them and the consumables used in direct patient care – A pharmacists gave input on the types of pharmaceuticals used for each intervention, as well as the average dosages and duration of treatment – A biomedical scientist gave input on the consumables used for laboratory services
Population in need	Mainly the Global Health Data Exchange, with additional data from the Ghana Statistical Service and academic literature	See details in Additional file 1: S3

#### Micro-costing healthcare interventions

Subsequently, a micro-costing exercise was performed, estimating cost per case treated in 2017 Ghana cedis (GH₵). Cost items included were: medicines, consumables, lodging costs during in-patient stays, and health worker wages. Medicine costs were largely taken from the NHIS Medicines List 2016 (and inflated to 2017 prices). The costs of medicines not covered through the NHIS were obtained through wholesale price quotes. Data on the costs of consumables were provided by the procurement departments of health facilities and by wholesale providers. In-patient lodging costs were quoted by a hospital. Health worker wages were obtained through Ministry of Health payroll data. We assumed that wages in non-government facilities are equal to wages in government facilities. Assumptions regarding the resources used per case treated for each intervention were based on the OneHealth Costing Tool and validated using expert opinion (see Table 1 for detail).

#### Estimating budget impact, net health benefit, and physician demand

The micro-costing exercise rendered healthcare costs per case treated. To calculate the budget impact for each healthcare intervention, costs per case treated were multiplied by the annual population in need of the intervention.

To evaluate interventions’ value for money, their incremental health benefits and costs are commonly combined into incremental cost-effectiveness ratios (ICERs). However, the alternative approach of using NHB (or, equivalently, net monetary benefit) is more suited for ranking large numbers of interventions and assessing the magnitude of the difference in benefit between interventions, and hence was used for this study [17]. The NHB of an intervention reflects its impact on total population health net of any health opportunity costs. To obtain the interventions’ annual NHB, their annual DALY and cost outcomes were combined with an estimate of the cost-effectiveness threshold [18, 19]. It was calculated using NHB = $${\Delta h}_{i}- {\Delta c}_{i}/k$$, where $${\Delta h}_{i}$$ = health gain (in DALYs avoided) of intervention *i*, $$\Delta {c}_{i}$$ = cost increase (in GH₵) of intervention *i*, and $$k$$ = the cost-effectiveness threshold (GH₵/DALY). Threshold value *k* reflects the opportunity cost of healthcare spending in Ghana. A 2018 study by Ochalek et al. provides estimates of the *k* threshold for a range of low-income countries [20]. In the study, the country-specific effects of healthcare expenditures on health outcomes (outcome elasticities) are estimated, using an instrumental variable approach to account for endogeneity. The estimated elasticities are subsequently applied to country-specific mortality and morbidity data to obtain the amount of money needed to avoid 1 DALY, i.e. the threshold value. The estimate of Ghana’s threshold is US$432 /DALY avoided [20]. Using the 2017 exchange rate of 4.4 GH₵/US$ [21], this translates to GH₵1880.

Finally, the full-time equivalent (FTE) of physicians needed for each intervention was estimated. It was assumed that physicians spend 30 h per week providing direct patientcare and work 46 weeks per year. To obtain the FTE physicians needed for each intervention, the total annual amount of physician hours needed for the intervention was divided by the annual hours available per physician.

### Step 2: assembling different options for the benefits package

Five different options for the benefits package were constructed, each inspired by current policy discourse on the design of the benefits package. The annual NHIS budget for the provision of the benefits package was assumed to be GH₵970 million (US$223 million), based on 2017 NHIS expenditure on claims reimbursement [23] See Additional file 1: S4 for details.

Table 2 details the assumptions that each package is based on. Package *Best buys* reflects the basic approach of the Ochalek et al. framework and aims to maximize value for money at the population level by prioritizing ‘best buys’: interventions with high NHB. This approach is in line with the objective stated by the Ghanaian Ministry of Health of increasing efficiency in the health sector [14]. Interventions are included in the benefits package in decreasing order of NHB, until the budget is exhausted [15]. As cost-effectiveness maximization has been the main paradigm in evidence-based priority-setting in health sectors around the world over recent years, prioritizing cost-effective interventions was assumed to be a secondary aim in all other packages. Package *Status quo* aims to reflect a continuation of the historical focus on service coverage (number of interventions included) at the expense of population coverage. A primary care package, which has often been coined as a first step in the direction of universal health coverage, is included [24]. Two options for a tiered system are also evaluated: one scenario in which only basic, community-level interventions are provided for free, and one in which only interventions with low budget impact are provided for free.

**Table 2:** Assumptions used for assembling the different benefits packages

Package	Primary goal	Co-payments for interventions included in package	Population coverage for interventions included in package
Best buys	Maximise value for money	No	100%
Status quo	Include high number of interventions	No	60%
Primary care	Include only primary care interventions	No	100%
Coinsurance—community care	Add co-insurance (for interventions that cannot be provided at community level by community health workers)	– No co-payments for community-level interventions – 50% coinsurance (with a cap at GH₵1,000) for interventions that are not provided at community-level	– 100% for interventions without coinsurance – 86% for interventions with coinsurance*
Coinsurance—budget	Add co-insurance (for interventions with high budget impact)	– No co-payments for interventions with budget impact < GH₵50 million – 50% coinsurance (with a cap at GH₵1,000) for interventions with budget impact ≥ GH₵50 million	– 100% for interventions without coinsurance – 86% for interventions with coinsurance*

*This figure is based on experimental research by Manning et al. [25], in which healthcare demand under a free healthcare plan is compared to healthcare demand under a 50% coinsurance plan.

In each of the five scenarios for the benefits package there were healthcare interventions that could not be included, as there are more healthcare interventions available than can fit within the annual NHIS budget. While excluded interventions can still be provided, their cost is fully borne by the healthcare user, through out-of-pocket payments. We assumed decreased healthcare demand (i.e. population coverage) for the interventions that were not included in the benefits package. We assumed healthcare demand to decrease from 100 to 78% for interventions not covered by the package, and from 100 to 86% for interventions with a 50% coinsurance rate [25] See Additional file 1: S5 for details.

### Step 3: outcomes for the five benefits packages

The outcome measures for the package scenarios closely follow the practical reality of policymakers by reflecting their various—sometimes contending—goals. Four outcome measures are presented: total annual NHB, the net physician availability, the number of interventions covered by the benefits package, and the annual number of cases treated through the NHIS.

‘Total annual NHB’ and ‘net physician availability’ are nation-wide outcomes. They indicate total annual gains in population health and total physician shortage in Ghana, in each scenario for the benefits package. ‘Total annual NHB’ (‘total FTE physicians required’) is calculated by adding together the aggregate NHB (FTE physicians required) of all the interventions included in the benefits package and the aggregate NHB (FTE physicians required) of all the interventions not included in the package (as mentioned above, reduced healthcare demand is assumed for the latter, which affects total NHB (FTE physicians required)). While relatively well-endowed with nurses, midwives and community health workers, Ghana is faced with a shortage of physicians, caused by low production levels and high levels of emigration among qualified practitioners [26]. According to the 2017 government payroll, there are 2,366 physicians working in the public health sector in Ghana. Given that 90% of health workers are estimated to work in the public sector [27], the total number of physicians in Ghana is assumed to be 2,627. ‘Net physician availability’ is defined as the number of full-time equivalent (FTE) physicians required in each package scenario, net of the 2627 physicians available. Given that the training of physicians takes time, benefits package scenarios that have a large physician shortage may be less feasible to fully implement in the short term.

‘Number of interventions included’ and ‘annual cases treated through the NHIS’ are benefits package-specific outcomes. While all five options for the benefits packages are aligned with the annually available budget, some packages cover a higher number of interventions than others. The addition of ‘number of interventions included’ to the outcome measures stems from worries among politicians about possible political backlash after reducing the number of interventions covered under the NHIS benefits package. ‘Annual cases treated through the NHIS’ reflects financial risk protection (very crudely): the higher the number of cases treated through the national health insurance scheme, the higher the number of patients avoiding having to pay their full treatment cost out-of-pocket. (It also might be interpreted as ‘number of potential voters experiencing access to insured care’ and as such be of interest to political actors). The ‘number of cases treated’ is calculated using the annual populations in need for the interventions included in each benefits package.

### Step 4: sensitivity analysis

Parameter uncertainty in the outcomes for the package scenarios was tested using one-way sensitivity analysis (SA). Two key input parameters are subject to methodological uncertainty: the cost-effectiveness threshold, and the price elasticity of healthcare demand. Two other input parameters relate to possible changes in the decision context: the NHIS budget, and the proportion of health worker wages borne by the NHIS. See Additional file 1: S6 for details.

## Results

For 69 interventions, sufficient data was available to estimate their health outcomes in Ghana. These interventions were included for consideration for the benefits package. Table 3 contains an overview of all interventions, including their population in need, as well as the budget impact, physician demand, and total NHB when the full population in need is treated. The interventions are listed in decreasing order of NHB, meaning that interventions higher on the list offer more value for money. Table 3 show that ‘good buys’ in Ghana are mostly in the areas of malaria, TB and maternal and neonatal care, as well as sexual reproductive health, child health and simple surgical procedures (inguinal hernia repair, cataract surgery). Worse buys are in cancer and other non-communicable diseases such as diabetes and cardiovascular disease. Worse buys in maternal and neonatal care and neglected tropical diseases appear to mostly be preventive drug treatments. Treatments for mental illnesses also perform poorly in terms of NHB.

**Table 3:** Interventions ranked according to net health benefit

#	Intervention*	Category	Provision level	Annual patient population	Budget impact (GH₵)	Total physician demand (FTE)**	Total NHB (DALYs avoided)
1	Drug treatment uncomplicated malaria in < 5 s	Malaria	Primary	3,358,476	63,724,968	0	12,821,265
2	Minimal DOTS plus resistant cases	Tuberculosis	Secondary and above	25,977	3,212,898	0	1,959,392
3	Full combination DOTS	Tuberculosis	Secondary and above	44,029	5,237,814	0	1,947,790
4	Full DOTS	Tuberculosis	Secondary and above	44,020	1,323,359	0	1,942,992
5	Minimal DOTS	Tuberculosis	Secondary and above	25,097	972,379	0	1,908,354
6	Emergency obstetric care	Maternal and neonatal	Secondary and above	35,432	45,713,781	14	931,631
7	Skilled maternal and immediate new-born care	Maternal and neonatal	Primary	876,577	79,109,064	318	621,643
8	Use of insecticide-treated bed nets	Malaria	Primary	3,774,440	7,169,971	0	563,514
9	Inguinal hernia repair	Surgical	Primary	59,447	16,084,990	0	544,304
10	Community-based support for low birthweight babies	Maternal and neonatal	Primary	43,595	113,245	0	448,821
11	Drug treatment sexually transmitted infections	Sexual and reproductive health	Primary	5,713,958	90,384,670	0	356,543
12	Voluntary Counselling and Testing	Sexual and reproductive health	Primary	125,524	1,690,712	0	284,050
13	Emergency neonatal care	Maternal and neonatal	Secondary and above	104,627	124,202,759	758	247,883
14	Antivenom for snakebites	Neglected tropical diseases	Primary	10,417	1,939,617	8	242,841
15	Oral rehydration solution for diarrhoea in < 5 s	Child health	Primary	7,928,521	20,898,714	0	224,820
16	Cataract surgery	Surgical	Primary	48,000	3,172,331	0	185,475
17	Tetanus toxoid vaccination (as part of antenatal care)	Maternal and neonatal	Primary	877,816	8,428,905	0	172,185
18	Drug treatment childhood pneumonia	Child health	Primary	1,095,649	8,846,996	0	159,424
19	Iron supplementation (pregnant women)	Maternal and neonatal	Primary	877,816	5,326,667	0	152,174
20	Syphilis detection and treatment (as part of antenatal care)	Maternal and neonatal	Primary	798,813	5,989,133	0	144,805
21	ART (first- and second-line treatment, intensive monitoring)	Sexual and reproductive health	Secondary and above	313,809	252,271,535	985	143,634
22	Antenatal corticosteroids for preterm labour	Maternal and neonatal	Primary	122,894	21,215,994	45	142,176
23	ART (first- and second-line treatment, no intensive monitoring)	Sexual and reproductive health	Secondary and above	280,208	214,576,816	271	139,873
24	Pre-referral rectal drug treatment malaria in < 5 s	Malaria	Primary	176,762	3,683,790	0	135,228
25	ART (first-line treatment, intensive monitoring)	Sexual and reproductive health	Secondary and above	219,667	124,887,298	690	132,020
26	ART (first-line treatment, no intensive monitoring)	Sexual and reproductive health	Primary	219,667	116,512,735	212	128,537
27	Community-based management of neonatal pneumonia	Maternal and neonatal	Primary	229,940	1,229,183	0	122,117
28	Male circumcision	Sexual and reproductive health	Primary	425,611	20,546,102	0	109,716
29	Antibiotics for pPROM	Maternal and neonatal	Primary	27,897	136,028	5	96,482
30	Case management of epilepsy	Psychological and neurological	Primary	137,757	19,285,785	33	66,738
31	Screening hearing loss	Ear, nose and throat	Primary	45,385	15,962,992	0	52,335
32	Intermittent preventive drug treatment malaria during pregnancy	Malaria	Primary	877,816	593,377	0	45,084
33	Heavy alcohol use, brief advice	Psychological and neurological	Primary	296,118	868,919	0	43,038
34	Pre-referral rectal drug treatment malaria in > 5 s	Malaria	Primary	50,014	1,042,305	0	41,638
35	Diabetes, retinopathy screening + photocoagulation	Non-communicable diseases	Primary	748,660	5,177,610	109	40,699
36	Screening children 5–15 for uncorrected refraction error	Child health	Primary	7,157,377	10,612,533	0	40,571
37	Iron supplementation in < 1 s	Child health	Primary	851,630	5,906,445	0	37,202
38	HPV [15, 17] vaccination	Non-communicable diseases	Primary	310,396	8,048,292	0	20,390
39	Integrated mass drug administration strategies for schistosomiasis and soil-transmitted helminthiasis (children 5–14 years old)	Neglected tropical diseases	Primary	6,560,411	28,538,496	0	18,406
40	Preventive drug treatment for patients at risk of post-partum haemorrhage	Maternal and neonatal	Primary	403,796	2,541,925	0	15,808
41	Integrated mass drug administration strategies for schistosomiasis and soil-transmitted helminthiasis (community-wide)	Neglected tropical diseases	Primary	28,308,301	141,276,210	0	13,006
42	Pap smear (at age 40) + treatment if necessary	Non-communicable diseases	Secondary and above	164,014	4,453,800	6	11,874
43	VIA (at age 40) + treatment if necessary	Non-communicable diseases	Secondary and above	164,014	5,301,245	6	11,691
44	Asymptotic bacteriuria detection and treatment (as part of antenatal care)	Maternal and neonatal	Primary	877,816	7,939,274	0	11,123
45	Drug treatment otitis media	Ear, nose and throat	Primary	2,035,324	18,202,098	0	10,281
46	VIA (at age 35,40,45) + treatment if necessary	Non-communicable diseases	Secondary and above	493,792	15,960,298	19	6378
47	Hepatitis B vaccination to prevent perinatal transmission	Maternal and neonatal	Primary	871,896	14,429,873	0	3383
48	Drug + psychosocial treatment schizophrenia	Psychological and neurological	Primary	43,170	16,210,167	16	3124
49	Isoniazid preventive therapy HIV-infected pregnant women	Maternal and neonatal	Primary	22,823	914,958	0	2056
50	Pap smear (at age 40) + removal of lesions	Non-communicable diseases	Primary	164,014	1,608,441	2	1469
51	VIA (at age 40) + removal of lesions	Non-communicable diseases	Primary	164,014	2,455,887	2	1316
52	Breast cancer, treatment stage I	Non-communicable diseases	Secondary and above	566	765,547	3	1202
53	Preventive drug treatment for patients at risk of CVD event	Non-communicable diseases	Primary	587,544	270,664,608	284	957
54	Isoniazid preventive therapy HIV-infected pregnant women with CD4 < 200	Maternal and neonatal	Primary	2282	91,496	0	174
55	VIA (at age 35,40,45) + removal of lesions	Non-communicable diseases	Primary	493,792	7,393,864	7	119
56	Breast cancer, treatment stage II	Non-communicable diseases	Secondary and above	850	2,597,700	7	− 548
57	Cervical cancer treatment	Non-communicable diseases	Secondary and above	8733	36,401,464	42	− 1309
58	Pap smear (every 5 years at ages 20–65) + removal of lesions	Non-communicable diseases	Primary	1,422,723	13,952,258	21	− 2774
59	Breast cancer, treatment stage IV	Non-communicable diseases	Secondary and above	1104	5,757,508	6	− 2944
60	Breast cancer, treatment stage III	Non-communicable diseases	Secondary and above	3476	15,686,449	39	− 5842
61	Breast cancer, treatment all stages	Non-communicable diseases	Secondary and above	5996	20,386,405	55	− 5899
62	Pap smear (every 5 years at ages 20–65) + treatment if necessary	Non-communicable diseases	Secondary and above	1,422,723	38,634,027	55	− 9707
63	Drug treatment of post-acute IHD & stroke	Non-communicable diseases	Primary	98,853	79,725,263	36	− 22,847
64	Drug treatment asthma	Non-communicable diseases	Primary	1,043,002	46,611,673	252	− 23,914
65	Drug + psychosocial treatment bipolar disorder	Psychological and neurological	Primary	154,857	89,753,143	56	− 26,123
66	Drug treatment bipolar disorder	Psychological and neurological	Primary	154,857	89,753,143	56	− 28,104
67	Episodic treatment unipolar depression	Psychological and neurological	Primary	809,667	146,767,346	196	− 39,527
68	Maintained drug + psychosocial treatment unipolar depression	Psychological and neurological	Primary	809,667	245,160,224	293	− 82,589
69	Diabetes, standard glycaemic control diabetes	Non-communicable diseases	Primary	748,660	2,686,931,378	99	− 1,391,459

*ART antiretroviral therapy, *CVD* cardiovascular disease, *DOTS* directly observed treatment, short course, *HPV* human papillomavirus, *IHD* ischaemic heart disease, *pPROM* preterm premature rupture of the membrane, *VIA* visual inspection of cervix with acetic acid

**When the physician demand is zero, this does not mean that no health workers are needed to provide these interventions but rather that the interventions can be provided by other health workers, such as community health workers, nurses and midwives

Implementing all interventions was estimated to cost GH₵4,323 million (US$994 million). (In cases of overlapping/mutually exclusive interventions, the one with highest NHB was selected. See Additional file 1: S7 for details). This is significantly more than the available budget of GH₵970 million (US$223 million). If the interventions are provided to only 40% of the population (approximating current coverage), total cost would be GH₵1,729 million (US$397 million).

### Comparing the packages

Tables 4 and 5 provides an overview of the included interventions in each package. Table 6 shows for each package scenario the total annual NHB, the net physician availability, the number of interventions included in the benefits package, and the number of cases treated through the NHIS.

**Table 4:** Interventions included in all packages

#	Category	Intervention
1	Child health	Drug treatment childhood pneumonia
2		Iron supplementation in < 1 s
3		Oral rehydration solution for diarrhoea in < 5 s
4		Screening children 5–15 for uncorrected refraction error
5	Ear, nose & throat	Drug treatment otitis media
6		Screening hearing loss
7	Malaria	Use of insecticide-treated bed nets
8		Pre-referral rectal drug treatment malaria in < 5 s
9		Pre-referral rectal drug treatment malaria in > 5 s
10		Drug treatment uncomplicated malaria in < 5 s
11	Maternal and neonatal	Antenatal corticosteroids for preterm labour
12		Antibiotics for pPROM
13		Asymptotic bacteriuria detection and treatment (as part of antenatal care)
14		Community-based management of neonatal pneumonia
15		Community-based support for low birthweight babies
16		Intermittent preventive drug treatment malaria during pregnancy
17		Iron supplementation (pregnant women)
18		Isoniazid preventive therapy HIV-infected pregnant women
19		Preventive drug treatment for patients at risk of post-partum haemorrhage
20		Skilled maternal and immediate new-born care
21		Syphilis detection and management (as part of antenatal care)
22		Tetanus toxoid vaccination (as part of antenatal care)
23	Neglected tropical diseases	Antivenom for snakebites
24		Integrated mass drug administration strategies for schistosomiasis and soil-transmitted helminthiasis (children 5–14 years old)
25	Non-communicable diseases	Diabetes, retinopathy screening + photocoagulation
26		HPV [15, 17] vaccination
27	Psychological and neurological	Case management of epilepsy
28		Heavy alcohol use, brief advice
29		Drug + psychosocial treatment schizophrenia
30	Sexual and reproductive health	Male circumcision
31		Drug treatment sexually transmitted infections
32		Voluntary Counselling and Testing
33	Surgical	Cataract surgery
34		Inguinal hernia repair

*pPROM preterm premature rupture of the membrane, *HPV* human papillomavirus

**Table 5:** Additional interventions included, per package

Category	Package *Best buys*	Package *Current*	Package *Primary care*	Package *Coinsurance—CHPS*	Package *Coinsurance—budget*
HIV/AIDS	ART (first- and second-line treatment, intensive monitoring)	ART (first- and second-line treatment, intensive monitoring)		ART (first- and second-line treatment, intensive monitoring)	ART (first- and second-line treatment, intensive monitoring)
			ART (first-line treatment, no intensive monitoring)		
Maternal and neonatal	Emergency neonatal care	Emergency neonatal care		Emergency neonatal care	Emergency neonatal care
	Emergency obstetric care	Emergency obstetric care		Emergency obstetric care	Emergency obstetric care
		Hepatitis B vaccination to prevent perinatal transmission	Hepatitis B vaccination to prevent perinatal transmission	Hepatitis B vaccination to prevent perinatal transmission	Hepatitis B vaccination to prevent perinatal transmission
Non-communicable diseases		Breast cancer treatment			Breast cancer treatment
		Cervical cancer treatment			Cervical cancer treatment
	Pap smear (at age 40) + treatment if necessary	Pap smear (at age 40) + treatment if necessary		Pap smear (at age 40) + treatment if necessary	Pap smear (at age 40) + treatment if necessary
			Pap smear (at age 40) + removal of lesions		
		Preventive drug treatment for patients at risk of CVD event	Preventive drug treatment for patients at risk of CVD event	Preventive drug treatment for patients at risk of CVD event	Preventive drug treatment for patients at risk of CVD event
		Drug treatment asthma		Drug treatment asthma	Drug treatment asthma
		Drug treatment of post-acute IHD & stroke	Drug treatment of post-acute IHD & stroke		Drug treatment of post-acute IHD & stroke
Psychological and neurological		Drug + psychosocial treatment bipolar disorder		Drug + psychosocial treatment bipolar disorder	Drug + psychosocial treatment bipolar disorder
		Episodic treatment unipolar depression		Episodic treatment unipolar depression	
Tuberculosis	Minimal DOTS plus resistant cases	Minimal DOTS plus resistant cases		Minimal DOTS plus resistant cases	Minimal DOTS plus resistant cases
			Minimal DOTS		

*ART antiretroviral therapy, *CVD* cardiovascular disease, *DOTS* directly observed treatment, short course, *IHD* ischaemic heart disease

**Table 6:** Outcomes per package

	Package	Total annual NHB (millions DALYs avoided)	Net physician availability (FTE)	Interventions included in the benefits package	Annual cases treated through the NHIS (millions)
1	Best buys	20.1	− 478	39	47.7
2	Status quo	18.2	− 387	47	30.7
3	Primary care	19.7	407	40	49.0
4	Coinsurance—community care	19.2	− 269	44	50.8
5	Coinsurance—budget	18.0	− 278	46	50.5

#### Best buys

As shown in Table 6, package *Best buys* renders the highest NHB, i.e. the best total population health. However, its number of included interventions is the lowest of all packages, due to the high budget impact of some of the more cost-effective interventions (e.g. emergency neonatal care, antiretroviral therapy for HIV/AIDS). Similarly, the package ranks last for net physician availability, as a result of the high physician demand among several of the ‘best buys’ interventions (e.g. skilled maternal and immediate new-born care, emergency neonatal care).

#### Status quo

Package *Status quo* performs poorly in terms of NHB as well as cases treated. This is explained by the inclusion of interventions that are not cost-effective and the relatively low population coverage. As a result of its prioritization of intervention coverage over population coverage, however, the package ranks first in terms of interventions included in the package.

#### Primary care

The *Primary care* package excludes several highly cost-effective interventions with high physician demand (most notably emergency neonatal care), as these are not provided at the primary care level. Resultingly, the package ranks lower than package *Best buys* for total NHB but performs much better in terms of net physician availability. Package *Primary care* also achieves a high number of cases treated through the NHIS.

#### Coinsurance—community care

Package *Coinsurance—community care* provides 28 interventions for free at the point of delivery. These are interventions that can be delivered by community health workers at community health centres. An additional 16 interventions are provided at 50% coinsurance. Compared to package *Primary care*, which focuses on non-specialized healthcare as well, package *Coinsurance—community care* includes more interventions and achieves a higher number of cases treated yet performs worse in terms of NHB and net physician availability. The average and median co-payments for the interventions that come with coinsurance are GH₵138 (around US$33) and GH₵33 (US$9), respectively (see Additional file 1: S8 for details).

#### Coinsurance—budget

38 interventions are included for free at the point of delivery in package *Coinsurance—budget*, while 8 interventions with high budget impact (> GH₵50 million, or US$11.5 million, at population-level) require co-payments. The package has a similar number of interventions included compared to package *Status quo*. While package *Coinsurance—budget* renders slightly less NHB than package *Status quo*, it performs better on ‘net physician availability’ and ‘cases treated’. The co-payments are a little higher for the interventions in this package, at an average of GH₵248 (US$57) and median of GH₵260 (US$60) (see Additional file 1: S8).

### Sensitivity analysis

Although the absolute values of the outcomes sometimes changed in responses to changes in the input parameters, the relative ranking of the packages on each outcome (e.g. packages with lowest and highest number of interventions included, respectively) remained virtually unchanged. Full SA results are available in Additional file 1: S9.

## Discussion

As argued previously, an explicit health benefits package is essential in creating a sustainable system for universal health coverage [28]. However, benefits packages in many Sub-Saharan countries, including Ghana, have historically been implicit, lacking an explicit overview of the included interventions and with limited alignment between healthcare aspirations and available resources [29]. With this study, we add to the literature by providing research results that can be used to define an explicit benefits package in Ghana. We made use of a framework that was developed in response to the lack of a widely accepted method for the development of benefits package [29]. We expanded upon the framework by evaluating different scenarios for the benefits package and by reporting multiple outcome measures. Herewith, we provide a practical illustration of one of the core elements for developing a benefits package (“Set goals & criteria”) that was identified in prior research by Glassman et al. [28].

Although decision making regarding the NHIS benefits package has historically been driven by political interests more than scientific evidence [12], recent years have seen an increased policy focus on data collection and analysis [14]. As discussions regarding a review of the NHIS benefits package are ongoing, this study provides a piece of evidence that can be referred to.

### Recommendations to policymakers in Ghana

The main aim of this study was to provide input into long-running discussions regarding the benefits package by making the trade-offs between different policy options explicit and transparent, without prescribing any single course of action. Further discussions and consensus-building between decisionmakers will be necessary to make unavoidable value judgements. Nonetheless, several general lessons emerge from the research.

Firstly, the current annual budget is insufficient to provide the large benefits package that the NHIS has historically claimed to provide. The inadequate resources for providing the full package are likely causing implicit rationing on the ground (e.g. individual healthcare providers may not provide certain medicines or charge out-of-pocket payments). This in turn leads to a prioritization of interventions and/or patients that may not be in line with government aims. If the government wishes to maintain the promise of a very large benefits package, resource allocation to the NHIS ought to be largely increased. If such increases to the NHIS are not made, it is recommended that the focus of the benefits package is moved from intervention coverage to population coverage. As shown by the low number of DALYs avoided for package *Status quo* (Table 6), focusing on intervention coverage at the expense of population coverage leads to unsatisfactory outcomes in terms of population health. The relatively positive outcomes for package *Coinsurance—community care* suggest that charging co-payments on specialized care could be a way to mitigate the apparent trade-off between population health and intervention coverage.

If policymakers wish to opt for a primary care package, it is recommended that provisions are made to include emergency obstetric and emergency neonatal care, despite being higher-level care, as these are highly cost-effective interventions. Given the high demand for specialist doctors of these interventions, it is recommended that efforts to train and retain relevant specialists are increased.

As shown in Table 3, interventions in the disease areas of malaria and tuberculosis are highly cost-effective. Given that some of the costs of managing these diseases are currently borne by disease-specific Control Programmes for which external funding is decreasing and projected to continue decreasing,[30] it is especially imperative for the Ghanaian government to ensure access to (cost-effective) malaria and TB interventions. Interventions in the area of non-communicable diseases tend to be less cost-effective. As shown in Table 3, various non-communicable diseases (NCD) interventions bring negative NHB, meaning that their implementation—at the expense of other interventions currently being provided—would decrease total population health. Since NCDs are strongly linked to lifestyle, further investigation of lifestyle interventions that would be feasible in Ghana would be useful.

The treatment of schizophrenia, unipolar depression and bipolar disorder were found to render low NHB. However, drug costs made up 98–99% of the total cost for these interventions. Policies to reduce drug prices for mental health interventions may therefore largely improve their cost-effectiveness.

### Limitations

This study was limited by data scarcity. Only interventions for which adequate information could be obtained on costs and health outcomes were included in the analysis, leaving out many possible interventions from consideration. Also, while financial risk protection is a key consideration when designing national health insurance schemes, it was deemed infeasible to incorporate this outcome measure into the analysis. Most existing methods require data on households’ financial resources per wealth/income quantile of the population [31]. Given Ghana’s large informal sector and limited data collection on the topic in population surveys, such data is not available. An alternative approach, which was used in the development of a health benefits package in Ethiopia, could be to assign scores for financial risk protection based on expert input [32]. More research on how financial risk protection can be evaluated in data-scarce settings may be valuable. Nonetheless, given that NHIS membership has been shown to improve financial risk protection, focusing on expanding current population coverage is likely to increase financial risk protection in the population.

While it would have been valuable to perform probabilistic sensitivity analysis, insufficient data was available. Nonetheless, the aim of this study was not to prescribe the exact package to be adopted by the NHIS and only general recommendations were given. Existing research supports these general recommendations, suggesting they are not very sensitive to uncertainty in the input parameters.

## Conclusions

In this study, the Ochalek et al. framework [15] was applied to the Ghanaian setting, in order to provide input into discussions regarding a review of the benefits package of the NHIS. The framework was adapted by adding a scenario analysis in which various packages were compiled based on discourse in Ghana’s health sector and by adding outcome measures beyond NHB. A key finding was that the size of the current package is not in line with the available budget. Given that decision-making with regards to Ghana’s NHIS benefits package tends to be driven by political interests, this study could be used to increase accountability among decisionmakers, and the same methods may be used to inform future decisions as the evidence base and budget evolve.

## Supplementary Information

**Additional file 1. S1.** Details of literature search on DALYs avoided. **S2.** References of cost-effectiveness studies included. **S3.** Data sources used to calculate annual population in need. **S4.** Calculating the annual budget available for claims reimbursement. **S5.** Calculating reduced healthcare demand. **S6.** Details on the sensitivity analysis. **S7.** Calculating the budget impact of implementing all interventions under consideration. **S8.** Co-payments for interventions in coinsurance packages. **S9.** Outcome tables for the sensitivity analysis.

## Data Availability

Further details about the analysis and additional results are made available in the Additional files. The micro-costing analysis can be made available upon request.

## Abbreviations

NHIS: National Health Insurance Scheme
NHIA: National Health Insurance Authority
HTA: Health technology assessment
NHB: Net health benefit
DALY: Disability-adjusted life-years
FTE: Full-time equivalent
SA: Sensitivity analysis
NCD: Non-communicable diseases
