# Supplementary material for: Supporting a review of the benefits package of the National Health Insurance Scheme in Ghana
Source: Cost Eff Resour Alloc. 2022 Jul 16;20:32. doi: 10.1186/s12962-022-00365-0 (PMC9287965; doi:10.1186/s12962-022-00365-0)
Supplement: Supplementary file 1 — Additional file 1. S1. Details of literature search on DALYs avoided. S2. References of cost-effectiveness studies included. S3. Data sources used to calculate annual population in need. S4. Calculating the annual budget available for claims reimbursement. S5. Calculating reduced healthcare demand. S6. Details on the sensitivity analysis. S7. Calculating the budget impact of implementing all interventions under consideration. S8. Co-payments for interventions in coinsurance packages. S9. Outcome tables for the sensitivity analysis. [file 12962_2022_365_MOESM1_ESM.docx]

**Additional file 1: S1 – Details of literature search on DALYs avoided**

Data on DALYs avoided was found through a literature search, using the Global Health CEA Registry. This is a comprehensive database of studies that use the ‘cost-per-DALY avoided’ metric to establish the efficacy of health interventions. Initially, only studies focusing on Sub-Saharan countries were considered for inclusion, by assessing the abstracts. Subsequently, for interventions that were still without DALY data after the first inclusion phase, any studies evaluating the intervention were considered regardless of their country setting.

The inclusion criteria comprised: research quality; applicability to Ghana; and availability of key information. The Global Health CEA Registry provides quality scorings, on a scale from 1(low) to 7(high). Studies with a score below 5 were generally excluded, though some cases were left to the researchers’ discretion. The applicability of each study to the Ghana context was decided based on two main factors: (i) the similarity in disease prevalence between Ghana and the country considered in the study, and (ii) the similarity in the income levels of the two countries. The disease prevalence was obtained using the GBD Results Tool. The income levels served as a proxy of the efficacy of the countries’ healthcare systems and were acquired through the World Bank DataBank.

The key information to be able to include the study comprised: a description of the population that had been treated; intervention coverage (i.e. the percentage of the ‘total population in need’ that received treatment); the comparator; and the total amount of DALYs avoided. These data items were extracted and subsequently the total DALYs avoided in Ghana were calculated for each intervention, assuming the intervention would be implemented at 100% coverage. Whenever a study assumed less than 100% coverage, its findings were extrapolated to 100%, assuming a linear increase in the DALYs avoided. Demographic and epidemiological data from the Ghana Statistical Service (GSS), Global Burden of Disease (GBD) Project, World Bank DataBank and a number of academic papers were used for adapting the studies’ findings to the Ghanaian context.

**Additional file 1: S2 – References of cost-effectiveness studies included**

Adam T, Lim SS, Mehta S, Bhutta ZA, Fogstad H, Mathai M, Zupan J, Darmstadt GL. Cost effectiveness analysis of strategies for maternal and neonatal health in developing countries. Bmj. 2005 Nov 10;331(7525):1107.

Baltussen R, Knai C, Sharan M. Iron fortification and iron supplementation are cost-effective interventions to reduce iron deficiency in four subregions of the world. The Journal of nutrition. 2004 Oct 1;134(10):2678-84.

Baltussen R, Floyd K, Dye C. Cost effectiveness analysis of strategies for tuberculosis control in developing countries. Bmj. 2005 Dec 8;331(7529):1364.

Baltussen R, Smith A. Cost effectiveness of strategies to combat vision and hearing loss in sub-Saharan Africa and South East Asia: mathematical modelling study. Bmj. 2012 Mar 2;344.

Buchanan J, Mihaylova B, Gray A, White N. Cost-effectiveness of pre-referral antimalarial, antibacterial, and combined rectal formulations for severe febrile illness. PLoS One. 2010 Dec 29;5(12):e14446.

Chisholm D, Saxena S. Cost effectiveness of strategies to combat neuropsychiatric conditions in sub-Saharan Africa and South East Asia: mathematical modelling study. Bmj. 2012 Mar 2;344.

Darmstadt GL, Bhutta ZA, Cousens S, Adam T, Walker N, De Bernis L, Lancet Neonatal Survival Steering Team. Evidence-based, cost-effective interventions: how many newborn babies can we save?. The Lancet. 2005 Mar 12;365(9463):977-88.

Edejer TT, Aikins M, Black R, Wolfson L, Hutubessy R, Evans DB. Cost effectiveness analysis of strategies for child health in developing countries. Bmj. 2005 Nov 17;331(7526):1177.

Ginsberg GM, Lauer JA, Zelle S, Baeten S, Baltussen R. Cost effectiveness of strategies to combat breast, cervical, and colorectal cancer in sub-Saharan Africa and South East Asia: mathematical modelling study. Bmj. 2012 Mar 2;344.

Gonzalez MA, Menendez C, Font F, Kahigwa E, Kimario J, Mshinda H, Tanner M, Bosch-Capblanch X, Alonso PL. Cost-effectiveness of iron supplementation and malaria chemoprophylaxis in the prevention of anaemia and malaria among Tanzanian infants. Bulletin of the World Health Organization. 2000;78:97-107.

Habib AG, Lamorde M, Dalhat MM, Habib ZG, Kuznik A. Cost-effectiveness of antivenoms for snakebite envenoming in Nigeria. PLoS neglected tropical diseases. 2015 Jan 8;9(1):e3381

Hogan DR, Baltussen R, Hayashi C, Lauer JA, Salomon JA. Cost effectiveness analysis of strategies to combat HIV/AIDS in developing countries. Bmj. 2005 Dec 15;331(7530):1431-7.

Kapoor S, Gupta A, Shah M. Cost-effectiveness of isoniazid preventive therapy for HIV-infected pregnant women in India. The International Journal of Tuberculosis and Lung Disease. 2016 Jan 1;20(1):85-92.

Kim JJ, Campos NG, O'Shea M, Diaz M, Mutyaba I. Model-based impact and cost-effectiveness of cervical cancer prevention in sub-Saharan Africa. Vaccine. 2013 Dec 29;31:F60-72.

Klingler C, Thoumi AI, Mrithinjayam VS. Cost-effectiveness analysis of an additional birth dose of Hepatitis B vaccine to prevent perinatal transmission in a medical setting in Mozambique. Vaccine. 2012 Dec 17;31(1):252-9.SS

Kuznik A, Lamorde M, Nyabigambo A, Manabe YC. Antenatal syphilis screening using point-of-care testing in Sub-Saharan African countries: a cost-effectiveness analysis. PLoS medicine. 2013 Nov 5;10(11):e1001545.

Lo NC, Bogoch II, Blackburn BG, Raso G, N'Goran EK, Coulibaly JT, Becker SL, Abrams HB, Utzinger J, Andrews JR. Comparison of community-wide, integrated mass drug administration strategies for schistosomiasis and soil-transmitted helminthiasis: a cost-effectiveness modelling study. The Lancet Global Health. 2015 Oct 1;3(10):e629-38.

Lubinga SJ, Atukunda EC, Wasswa-Ssalongo G, Babigumira JB. Potential cost-effectiveness of prenatal distribution of misoprostol for prevention of postpartum hemorrhage in Uganda. PloS one. 2015 Nov 11;10(11):e0142550.

Morel CM, Lauer JA, Evans DB. Cost effectiveness analysis of strategies to combat malaria in developing countries. Bmj. 2005 Dec 1;331(7528):1299.

Mori AT, Ngalesoni F, Norheim OF, Robberstad B. Cost-effectiveness of dihydroartemisinin-piperaquine compared with artemether-lumefantrine for treating uncomplicated malaria in children at a district hospital in Tanzania. Malaria journal. 2014 Dec;13(1):1-3.

Ortegón M, Lim S, Chisholm D, Mendis S. Cost effectiveness of strategies to combat cardiovascular disease, diabetes, and tobacco use in sub-Saharan Africa and South East Asia: mathematical modelling study. Bmj. 2012 Mar 2;344.

Seo MK, Baker P, Ngo KN. Cost-effectiveness analysis of vaccinating children in Malawi with RTS, S vaccines in comparison with long-lasting insecticide-treated nets. Malaria journal. 2014 Dec;13(1):1-1.

Shillcutt, S.D., Clarke, M.G. and Kingsnorth, A.N., 2010. Cost-effectiveness of groin hernia surgery in the Western Region of Ghana. Archives of surgery, 145(10), pp.954-961.

Stanciole AE, Ortegón M, Chisholm D, Lauer JA. Cost effectiveness of strategies to combat chronic obstructive pulmonary disease and asthma in sub-Saharan Africa and South East Asia: mathematical modelling study. Bmj. 2012 Mar 2;344.

Uthman OA, Popoola TA, Yahaya I, Uthman MM, Aremu O. The cost-utility analysis of adult male circumcision for prevention of heterosexual acquisition of HIV in men in sub-Saharan Africa: a probabilistic decision model. Value in Health. 2011 Jan 1;14(1):70-9.

**Additional file 1: S3 – Data sources used to calculate annual population in need**

The estimated yearly population in need was based on: *size of the target group* for preventive interventions; *prevalence* for the treatment of chronic conditions; and *incidence* for the treatment of non-chronic conditions.

| **#** | **Intervention** | **Description patient population** | **Data source** |
| --- | --- | --- | --- |
| 1 | Drug treatment uncomplicated malaria in <5s | Incidence uncomplicated malaria | - *incidence malaria in <5s* GHDx (2016)  - *% severe malaria* Buchanan et al. (2010) |
| 2 | Minimal DOTS plus resistant cases | Incidence smear-positive + multi-drug resistant cases | - *incidence tuberculosis* GHDx (2016)  - *% smear-positive*, *% multi-drug resistant* Baltussen et al. (2005) |
| 3 | Full combination DOTS | Incidence smear-positive + smear-negative + extra-pulmonary + multi-drug resistant cases | - *incidence tuberculosis* GHDx (2016) |
| 4 | Full DOTS | Incidence smear-positive + smear-negative + extra-pulmonary cases | - *incidence tuberculosis* GHDx (2016)  - *% multi-drug resistant* Baltussen et al. (2005) |
| 5 | Minimal DOTS | Incidence smear-positive cases | - *incidence tuberculosis* GHDx (2016)  - *% smear-positive* Baltussen et al. (2005) |
| 6 | Emergency obstetric care | Incidence obstructed labour + severe pre-eclampsia + maternal sepsis | GHDx (2016) |
| 7 | Skilled maternal and immediate new-born care | Live births and still births | - *total population* GSS (2018)  - *live birth rate* GSS (2014)  - *still birth rate* MoH (2018) |
| 8 | Use of insecticide-treated bed nets | Total population | - *total population* GSS (2018)  - *years that bed nets last* Morel et al. (2015)  - *persons per bed net* Morel et al. (2015) |
| 9 | Inguinal hernia repair | Incidence inguinal hernia | Beard et al. (2013) |
| 10 | Community-based support for low birthweight babies | Low birthweight babies | - *total population* GSS (2018)  - *live birth rate* GSS (2014)  - *% of births with low birthweight* Darmstadt et al. (2005) |
| 11 | Drug treatment sexually transmitted infections | Prevalence STIs | GHDx (2016) |
| 12 | Voluntary Counselling and Testing | Over 5-year period twice annual prevalence | GHDx (2016) |
| 13 | Emergency neonatal care | Incidence severe neonatal infections + neonatal jaundice + severe asphyxia + low birthweight | - *total population* GSS (2018)  - *live birth rate* GSS (2014)  - *% of new-borns that needs ENC* Darmstadt et al. (2005) |
| 14 | Antivenom for snakebites | Incidence of venomous snake bites | - *total population* GSS (2018)  - *% snake bites* Punguyire et al. (2014)  - *% of snake bits that is venomous* Habib et al. (2015) |
| 15 | Oral rehydration solution for diarrhoea in <5s | Incidence diarrheal diseases | GHDx (2016) |
| 16 | Cataract surgery | Incidence cataract blindness | National Eye Care Unit (2008) |
| 17 | Tetanus toxoid vaccination (as part of antenatal care) | Pregnant women | - *total population* GSS (2018)  - *live birth rate* GSS (2014)  - *still birth rate* MoH (2018)  - *institutional maternal mortality rate* MoH (2018) |
| 18 | Drug treatment childhood pneumonia | Incidence childhood pneumonia | Walker et al. (2013) |
| 19 | Iron supplementation (pregnant women) | Pregnant women | - *total population* GSS (2018)  - *live birth rate* GSS (2014)  - *still birth rate* MoH (2018)  - *institutional maternal mortality rate* MoH (2018) |
| 20 | Syphilis detection and treatment (as part of antenatal care) | Pregnant women | - *total population* GSS (2018)  - *live birth rate* GSS (2014)  - *still birth rate* MoH (2018)  - *institutional maternal mortality rate* MoH (2018) |
| 21 | ART (first- and second-line treatment, intensive monitoring) | Prevalence HIV | GHDx (2016) |
| 22 | Antenatal corticosteroids for preterm labour | Incidence pre-term labour | Nkyekyer et al. (2006) |
| 23 | ART (first- and second-line treatment, no intensive monitoring) | Prevalence HIV | GHDx (2016) |
| 24 | Pre-referral rectal drug treatment malaria in <5s | Incidence severe malaria | - *incidence malaria in <5s* GHDx (2016)  - *% severe malaria* Buchanan et al. (2010) |
| 25 | ART (first-line treatment, intensive monitoring) | Number on first-line treatment | - *prevalence HIV* GHDx (2016)  - *% on first-line treatment* Hogan et al. (2005) |
| 26 | ART (first-line treatment, no intensive monitoring) | Number on first-line treatment | - *prevalence HIV* GHDx (2016)  - *% on first-line treatment* Hogan et al. (2005) |
| 27 | Community-based management of neonatal pneumonia | Incidence neonatal pneumonia | Walker et al. (2013) |
| 28 | Male circumcision | Boys < 1 | GSS (2018) |
| 29 | Antibiotics for pPROM | Incidence pPROM | Nkyekyer et al. (2006) |
| 30 | Case management of epilepsy | Prevalence of epilepsy | GHDx (2016) |
| 31 | Screening hearing loss | Prevalence hearing impairment | GHDx (2016) |
| 32 | Intermittent preventive drug treatment malaria during pregnancy | Pregnant women | - *total population* GSS (2018)  - *live birth rate* GSS (2014)  - *still birth rate* MoH (2018)  - *institutional maternal mortality rate* MoH (2018) |
| 33 | Heavy alcohol use, brief advice | Prevalence heavy alcohol use | GHDx (2016) |
| 34 | Pre-referral rectal drug treatment malaria in >5s | Incidence severe malaria | - *incidence malaria in <5s* GHDx (2016)  - *% severe malaria* Buchanan et al. (2010) |
| 35 | Diabetes, retinopathy screening + photocoagulation | Prevalence of diabetes | GHDx (2016) |
| 36 | Screening children 5-15 for uncorrected refraction error | Children 5-15 | GSS (2016) |
| 37 | Iron supplementation in <1s | Infants < 1 | GSS (2016) |
| 38 | HPV (16,18) vaccination | Girls aged 12 | GSS (2016) |
| 39 | Integrated mass drug administration strategies for schistosomiasis and soil-transmitted helminthiasis (children 5-14 years old) | Children 5-14 | GSS (2016) |
| 40 | Preventive drug treatment for patients at risk of post-partum haemorrhage | Pregnant women | - *total population* GSS (2018)  - *live birth rate* GSS (2014)  - *still birth rate* MoH (2018)  - *institutional maternal mortality rate* MoH (2018) |
| 41 | Integrated mass drug administration strategies for schistosomiasis and soil-transmitted helminthiasis (community-wide) | Total population | GSS (2016) |
| 42 | Pap smear (at age 40) + treatment if necessary | Women aged 40 | GSS (2016) |
| 43 | VIA (at age 40) + treatment if necessary | Women aged 40 | GSS (2016) |
| 44 | Asymptotic bacteriuria detection and treatment (as part of antenatal care) | Pregnant women | - *total population* GSS (2018)  - *live birth rate* GSS (2014)  - *still birth rate* MoH (2018)  - *institutional maternal mortality rate* MoH (2018) |
| 45 | Drug treatment otitis media | Incidence otitis media | GHDx (2016) |
| 46 | VIA (at age 35,40,45) + treatment if necessary | Women aged 35, 40, 45 | GSS (2016) |
| 47 | Hepatitis B vaccination to prevent perinatal transmission | Live births | - *total population* GSS (2018)  - *live birth rate* GSS (2014) |
| 48 | Drug + psychosocial treatment schizophrenia | Prevalence schizophrenia | GHDx (2016) |
| 49 | Isoniazid preventive therapy HIV-infected pregnant women | Prevalence HIV among pregnant women | MoH (2018) |
| 50 | Pap smear (at age 40) + removal of lesions | Women aged 40 | GSS (2016) |
| 51 | VIA (at age 40) + removal of lesions | Women aged 40 | GSS (2016) |
| 52 | Breast cancer, treatment stage I | Prevalence of stage I breast cancer | - *prevalence breast cancer* GHDx (2016)  - *% of stage I* Groot et al. (2006) |
| 53 | Preventive drug treatment for patients at risk of CVD event | Prevalence of people at risk of CVD event | Boateng et al. (2017) |
| 54 | Isoniazid preventive therapy HIV-infected pregnant women with CD4 < 200 | Prevalence HIV with CD4 < 200 | - *prevalence HIV* GHDx (2016)  - *% with CDV4 < 200* Kapoor et al. (2016) |
| 55 | VIA (at age 35,40,45) + removal of lesions | Women aged 35, 40, 45 | GSS (2016) |
| 56 | Breast cancer, treatment stage II | Prevalence of stage II breast cancer | - *prevalence breast cancer* GHDx (2016)  - *% of stage I* Groot et al. (2006) |
| 57 | Cervical cancer treatment | Prevalence of cervical cancer | GHDx (2016) |
| 58 | Pap smear (every 5 years at ages 20-65) + removal of lesions | Women aged 20-65 (once every 5 years) | GSS (2016) |
| 59 | Breast cancer, treatment stage IV | Prevalence of stage IV breast cancer | - *prevalence breast cancer* GHDx (2016)  - *% of stage I* Groot et al. (2006) |
| 60 | Breast cancer, treatment stage III | Prevalence of stage III breast cancer | - *prevalence breast cancer* GHDx (2016)  - *% of stage I* Groot et al. (2006) |
| 61 | Breast cancer, treatment all stages | Prevalence of breast cancer | GHDx (2016) |
| 62 | Pap smear (every 5 years at ages 20-65) + treatment if necessary | Women aged 20-65 (once every 5 years) | GSS (2016) |
| 63 | Drug treatment of post-acute IHD & stroke | Prevalence of post-acute IHD and stroke | GHDx (2016) |
| 64 | Drug treatment asthma | Prevalence (mild persistent) asthma | GHDx (2016) |
| 65 | Drug + psychosocial treatment bipolar disorder | Prevalence bipolar disorder | GHDx (2016) |
| 66 | Drug treatment bipolar disorder | Prevalence bipolar disorder | GHDx (2016) |
| 67 | Episodic treatment unipolar depression | Prevalence depression | GHDx (2016) |
| 68 | Maintained drug + psychosocial treatment unipolar depression | Prevalence depression | GHDx (2016) |
| 69 | Diabetes, standard glycaemic control diabetes | Prevalence of diabetes | GHDx (2016) |

***References***

Baltussen R, Floyd K, Dye C. Cost effectiveness analysis of strategies for tuberculosis control in developing countries. Bmj. 2005 Dec 8;33

Beard JH, Oresanya LB, Ohene-Yeboah M, Dicker RA, Harris HW. Characterizing the global burden of surgical disease: a method to estimate inguinal hernia epidemiology in Ghana. World journal of surgery. 2013 Mar;37(3):498-503.

Boateng D, Agyemang C, Beune E, Meeks K, Smeeth L, Schulze M, Addo J, de-Graft Aikins A, Galbete C, Bahendeka S, Danquah I. Migration and cardiovascular disease risk among ghanaian populations in Europe: the RODAM Study (Research on Obesity and Diabetes Among African Migrants). Circulation: Cardiovascular Quality and Outcomes. 2017 Nov;10(11):e004013.

Buchanan J, Mihaylova B, Gray A, White N. Cost-effectiveness of pre-referral antimalarial, antibacterial, and combined rectal formulations for severe febrile illness. PLoS One. 2010 Dec 29;5(12):e14446.

Darmstadt GL, Bhutta ZA, Cousens S, Adam T, Walker N, De Bernis L, Lancet Neonatal Survival Steering Team. Evidence-based, cost-effective interventions: how many newborn babies can we save?. The Lancet. 2005 Mar 12;365(9463):977-88.

Global Health Data Exchange (GHDx). GBD Results Tool. 2016. Available at: <http://ghdx.healthdata.org/gbd-results-tool>.

Ghana Statistical Service (GSS). 2010 Population & Housing Census Report: Fertility. 2014.

Ghana Statistical Service (GSS). Population projections 2010-2020. 2018. Unpublished.

Groot MT, Baltussen R, Uyl‐de Groot CA, Anderson BO, Hortobágyi GN. Costs and health effects of breast cancer interventions in epidemiologically different regions of Africa, North America, and Asia. The breast journal. 2006 Jan;12:S81-90.

Habib AG, Lamorde M, Dalhat MM, Habib ZG, Kuznik A. Cost-effectiveness of antivenoms for snakebite envenoming in Nigeria. PLoS neglected tropical diseases. 2015 Jan 8;9(1):e3381

Hogan DR, Baltussen R, Hayashi C, Lauer JA, Salomon JA. Cost effectiveness analysis of strategies to combat HIV/AIDS in developing countries. Bmj. 2005 Dec 15;331(7530):1431-7.

Kapoor S, Gupta A, Shah M. Cost-effectiveness of isoniazid preventive therapy for HIV-infected pregnant women in India. The International Journal of Tuberculosis and Lung Disease. 2016 Jan 1;20(1):85-92.

Ministry of Health (MoH). Holistic Assessment of 2017 Health Sector Programme of Work. 2018.

Morel CM, Lauer JA, Evans DB. Cost effectiveness analysis of strategies to combat malaria in developing countries. Bmj. 2005 Dec 1;331(7528):1299.

Nkyekyer K, Laryea C, Boafor T. Singleton preterm births in Korle bu teaching hospital, Accra, Ghana–origins and outcomes. Ghana Medical Journal. 2006;40(3).

Punguyire D, Baiden F, Nyuzaghl J, Hultgren A, Berko Y, Brenner S, Soghoian S, Adjei G, Niyogi A, Moresky R. Presentation, management, and outcome of snake-bite in two district hospitals in Ghana. The Pan African medical journal. 2014;19(219).

Walker CL, Rudan I, Liu L, Nair H, Theodoratou E, Bhutta ZA, O'Brien KL, Campbell H, Black RE. Global burden of childhood pneumonia and diarrhoea. The Lancet. 2013 Apr 20;381(9875):1405-16.

**Additional file 1: S4 – Calculating the annual budget available for claims reimbursement**

The estimated budget was based on the 2017 NHIS expenditure on claims reimbursement, which was GH₵1,396 million (US$321 million). However, NHIS payments to providers are intended to cover direct costs as well as overhead costs, while only direct costs are included in our analysis (i.e. medicines, consumables, in-patient days and wages for healthcare providers). The figure for 2017 expenditure on claims reimbursement was therefore adjusted using an estimate of healthcare facilities’ overhead costs.

In a 2010 study, Aboagye et al. estimated the overhead costs in three different types of health centers: referral, district and mission hospitals. The weighted average was estimated to be 30%, using the national distribution of facilities as quoted by Aboagye et al.

| **Type of facility** | **Number of facilities** | **Overhead costs as a percentage of total cost** |
| --- | --- | --- |
| Mission hospital | 40 | 20% |
| District hospital | 70 | 35% |
| Referral hospital | 9 | 42% |

*Source: Aboagye AQ, Degboe AN, Obuobi AA. Estimating the cost of healthcare delivery in three hospitals in southern ghana. Ghana Med J. 2010;44(3):83-92.*

The annual NHIS budget available for covering the costs included in our analysis was therefore estimated to be GH₵970 million (US$223 million)

**Additional file 1: S5 – Calculating reduced healthcare demand**

The results from the Rand Health Insurance Experiment in the United States, reported on in the 1987 Manning et al. study, are the most widely used and accepted estimates of the price elasticity of healthcare demand. For this analysis, the results in their Table 3 were used, which show the estimated likelihood of any healthcare use under different insurance plans.

| **NHIS arrangement** | **Insurance plan in Manning et al. (1987)** | **Likelihood of any use of medical services according to Table 3 in Manning et al. (1987)** | **Calculation to obtain estimate of reduction in healthcare demand per NHIS arrangements** | **Assumed healthcare demand per NHIS arrangement** |
| --- | --- | --- | --- | --- |
| 0% coinsurance, healthcare free at the point of delivery | Free | 86.7% |  | 100% |
| 50% coinsurance | 50% coinsurance | 74.3% | = (74.3 – 86.7) / 86.7 | 86% |
| 100% coinsurance,  healthcare not covered | 95% coinsurance | 68.0% | = (68.0 – 86.7) / 86.7 | 78% |

*Source: Manning WG, Newhouse JP, Duan N, Keeler EB, Leibowitz A, Marquis MS. Health insurance and the demand for medical care: evidence from a randomized experiment. Am Econ Rev. 1987;77(3):251-77.*

**Additional file 1: S6 – Details on the sensitivity analysis**

For the SA on the cost-effectiveness threshold, the minimum and maximum estimated values for the threshold in Ghana were used.(21) Since no suitable research on the price elasticity of healthcare demand in Ghana was available, the effect of a range of possible values was evaluated.

The effect of an increase as well as a decrease in the NHIS budget was evaluated. The value for a decreased NHIS budget was calculated by subtracting the annual NHIS deficit of approximately GH₵300 million from the base case budget. The value for an increased budget was calculated by assuming that the percentage of claims expenditure increases from the current 77% of total expenditure to the target of 85% (implying improved efficiency in the administration of the scheme).

Currently, only health worker wages in private health facilities are paid using NHIS funding, while health worker wages in faith-based and government facilities are paid through the central government roll. In our base case, health worker costs for faith-based and government facilities were therefore excluded in the cost calculations. However, the detachment of paying (and promoting) health workers from the daily management of the facilities where they work, is commonly cited as a contributing factor to inefficiency in the health sector. To assess the effect of a policy change that would render the individual facilities responsible for paying their workers through NHIS funds, a scenario in which all health worker costs are paid through the NHIS is included in the SA. Note that this SA could also be seen to reflect uncertainty around our assumption that wages in non-government facilities are equal to wages in government facilities.

**Additional file 1: S7 – Calculating the budget impact of implementing all interventions under consideration**

| **#** | **Intervention** | **Budget impact (GH₵)**  ***100% population coverage*** | **Budget impact (GH₵)**  ***40% population coverage*** |
| --- | --- | --- | --- |
| 1 | Drug treatment uncomplicated malaria in <5s | 63,724,968 | 25,489,987 |
| 2 | Minimal DOTS plus resistant cases | 3,212,898 | 1,285,159 |
| 3 | Emergency obstetric care | 45,713,781 | 18,285,512 |
| 4 | Skilled maternal and immediate new-born care | 79,109,064 | 31,643,626 |
| 5 | Use of insecticide-treated bed nets | 7,169,971 | 2,867,989 |
| 6 | Inguinal hernia repair | 16,084,990 | 6,433,996 |
| 7 | Community-based support for low birthweight babies | 113,245 | 45,298 |
| 8 | Drug treatment sexually transmitted infections | 90,384,670 | 36,153,868 |
| 9 | Voluntary Counselling and Testing | 1,690,712 | 676,285 |
| 10 | Emergency neonatal care | 124,202,759 | 49,681,104 |
| 11 | Antivenom for snakebites | 1,939,617 | 775,847 |
| 12 | Oral rehydration solution for diarrhoea in <5s | 20,898,714 | 8,359,485 |
| 13 | Cataract surgery | 3,172,331 | 1,268,932 |
| 14 | Tetanus toxoid vaccination (as part of antenatal care) | 8,428,905 | 3,371,562 |
| 15 | Drug treatment childhood pneumonia | 8,846,996 | 3,538,798 |
| 16 | Iron supplementation (pregnant women) | 5,326,667 | 2,130,667 |
| 17 | Syphilis detection and treatment (as part of antenatal care) | 5,989,133 | 2,395,653 |
| 18 | ART (first- and second-line treatment, intensive monitoring) | 252,271,535 | 100,908,614 |
| 19 | Antenatal corticosteroids for preterm labour | 21,215,994 | 8,486,398 |
| 20 | Pre-referral rectal drug treatment malaria in <5s | 3,683,790 | 1,473,516 |
| 21 | Community-based management of neonatal pneumonia | 1,229,183 | 491,673 |
| 22 | Male circumcision | 20,546,102 | 8,218,441 |
| 23 | Antibiotics for pPROM | 136,028 | 54,411 |
| 24 | Case management of epilepsy | 19,285,785 | 7,714,314 |
| 25 | Screening hearing loss | 15,962,992 | 6,385,197 |
| 26 | Intermittent preventive drug treatment malaria during pregnancy | 593,377 | 237,351 |
| 27 | Heavy alcohol use, brief advice | 868,919 | 347,567 |
| 28 | Pre-referral rectal drug treatment malaria in >5s | 1,042,305 | 416,922 |
| 29 | Diabetes, retinopathy screening + photocoagulation | 5,177,610 | 2,071,044 |
| 30 | Screening children 5-15 for uncorrected refraction error | 10,612,533 | 4,245,013 |
| 31 | Iron supplementation in <1s | 5,906,445 | 2,362,578 |
| 32 | HPV (16,18) vaccination | 8,048,292 | 3,219,317 |
| 33 | Integrated mass drug administration strategies for schistosomiasis and soil-transmitted helminthiasis (children 5-14 years old) | 28,538,496 | 11,415,398 |
| 34 | Preventive drug treatment for patients at risk of post-partum haemorrhage | 2,541,925 | 1,016,770 |
| 35 | Pap smear (at age 40) + cancer treatment if necessary | 4,453,800 | 1,781,520 |
| 36 | Asymptotic bacteriuria detection and treatment (as part of antenatal care) | 7,939,274 | 3,175,710 |
| 37 | Drug treatment otitis media | 18,202,098 | 7,280,839 |
| 38 | Hepatitis B vaccination to prevent perinatal transmission | 14,429,873 | 5,771,949 |
| 39 | Drug + psychosocial treatment schizophrenia | 16,210,167 | 6,484,067 |
| 40 | Isoniazid preventive therapy HIV-infected pregnant women | 914,958 | 365,983 |
| 41 | Preventive drug treatment for patients at risk of CVD event | 270,664,608 | 108,265,843 |
| 42 | Cervical cancer treatment | 36,401,464 | 14,560,586 |
| 43 | Breast cancer, treatment all stages | 20,386,405 | 8,154,562 |
| 44 | Drug treatment of post-acute IHD & stroke | 79,725,263 | 31,890,105 |
| 45 | Drug treatment asthma | 46,611,673 | 18,644,669 |
| 46 | Drug + psychosocial treatment bipolar disorder | 89,753,143 | 35,901,257 |
| 47 | Episodic treatment unipolar depression | 146,767,346 | 58,706,938 |
| 48 | Diabetes, standard glycaemic control diabetes | 2,686,931,378 | 1,074,772,551 |
|  | **TOTAL** | **4,323,062,183** | **1,729,224,873** |
|  |  |  |  |

**Additional file 1: S8 – Co-payments for interventions in coinsurance packages**

Co-payments for interventions in Package *Coinsurance – community care*

| **#** | **Intervention** | **Co-payment**  **(GH₵)** |
| --- | --- | --- |
| 1 | Antibiotics for pPROM | 2 |
| 2 | Diabetes, retinopathy screening + photocoagulation | 3 |
| 3 | Syphilis detection and management (as part of antenatal care) | 4 |
| 4 | Voluntary Counselling and Testing | 7 |
| 5 | Drug treatment sexually transmitted infections | 8 |
| 6 | Pap smear (at age 40) + cervical cancer treatment if necessary | 14 |
| 7 | Male circumcision | 24 |
| 8 | Cataract surgery | 33 |
| 9 | Skilled maternal and immediate new-born care | 45 |
| 10 | Minimal DOTS plus resistant cases | 62 |
| 11 | Antenatal corticosteroids for preterm labour | 86 |
| 12 | Inguinal hernia repair | 135 |
| 13 | Preventive drug treatment for patients at risk of a cardiovascular disease (CVD) event | 230 |
| 14 | ART (first- and second-line treatment, intensive monitoring) | 402 |
| 15 | Emergency neonatal care | 594 |
| 16 | Emergency obstetric care | 645 |
| AVERAGE CO-PAYMENT | | 143 |
| MEDIAN CO-PAYMENT | | 39 |

*pPROM = preterm premature rupture of the membrane,* *DOTS = directly observed treatment, short course, ART = antiretroviral therapy.*

Co-payments for interventions in Package *Coinsurance – budget*

| **#** | **Intervention** | **Co-payment**  **(GH₵)** |
| --- | --- | --- |
| 1 | Drug treatment sexually transmitted infections | 8 |
| 2 | Treatment uncomplicated malaria in <5s | 9 |
| 3 | Skilled maternal and immediate new-born care | 45 |
| 4 | Preventive drug treatment for patients at risk of CVD event | 230 |
| 5 | Drug + psychosocial treatment bipolar disorder | 290 |
| 6 | ART (first- and second-line treatment, intensive monitoring) | 402 |
| 7 | Drug treatment of post-acute IHD & stroke | 403 |
| 8 | Emergency neonatal care | 594 |
| AVERAGE CO-PAYMENT | | 248 |
| MEDIAN CO-PAYMENT | | 260 |

*CVD = cardiovascular disease, ART = antiretroviral therapy, IHD = ischaemic heart disease.*

**Additional file 1: S9 – Outcome tables for the sensitivity analysis**

**Varying the cost-effectiveness threshold:**

|  | **Package** | **Total annual NHB (millions DALYs avoided)** | | | **Net physician availability (FTE)** | | | **Interventions included in the benefits package** | | | **Annual cases treated through the NHIS (millions)** | | |
| --- | --- | --- | --- | --- | --- | --- | --- | --- | --- | --- | --- | --- | --- |
|  |  | **Base case** | **Lowest threshold** | **Highest threshold** | **Base case** | **Lowest threshold** | **Highest threshold** | **Base case** | **Lowest threshold** | **Highest threshold** | **Base case** | **Lowest threshold** | **Highest threshold** |
| ***1*** | ***Best buys*** | **20.1** | 19.8 | 20.3 | **-478** | -478 | -484 | **39** | 40 | 33 | **47.7** | 47.8 | 44.2 |
| ***2*** | ***Status quo*** | **18.2** | 17.9 | 18.5 | **-387** | -387 | -387 | **47** | 47 | 47 | **30.7** | 30.7 | 30.7 |
| ***3*** | ***Primary care*** | **19.7** | 19.4 | 20.0 | **407** | 358 | 407 | **40** | 40 | 40 | **49.0** | 50.0 | 49.0 |
| ***4A*** | ***Coinsurance – community care*** | **19.2** | 18.9 | 19.5 | **-269** | -270 | -269 | **44** | 45 | 43 | **50.8** | 50.7 | 50.7 |
| ***4B*** | ***Coinsurance – budget*** | **18.0** | 17.7 | 18.3 | **-278** | -278 | -278 | **46** | 46 | 46 | **50.5** | 50.5 | 50.5 |

**Varying the demand elasticity**:**

|  | **Package** | **Total annual NHB (millions DALYs avoided)** | | | | | | | | **Net physician availability (FTE)** | | | | | | | |
| --- | --- | --- | --- | --- | --- | --- | --- | --- | --- | --- | --- | --- | --- | --- | --- | --- | --- |
|  |  | **Base case**  **(14% / 22%)** | **Base case * 2 (29% / 43%)** | **5% / 10%** | **10% / 20%** | **15% / 30%** | **20% / 40%** | **25% / 50%** | **30% / 60%** | **Base case**  **(14% / 22%)** | **Base case * 2**  **(29% / 43%)** | **5% / 10%** | **10% / 20%** | **15% / 30%** | **20% / 40%** | **25% / 50%** | **30% / 60%** |
| ***1*** | ***Best buys*** | **20.1** | 20.4 | 20.0 | 20.1 | 20.2 | 20.4 | 20.5 | 20.7 | **-478** | -266 | -591 | -493 | -395 | -297 | -199 | -101 |
| ***2*** | ***Status quo*** | **18.2** | 16.7 | 19.0 | 18.3 | 17.6 | 16.9 | 16.2 | 15.5 | **-387** | -84 | -549 | -408 | -268 | -128 | 12 | 152 |
| ***3*** | ***Primary care*** | **19.7** | 19.8 | 19.7 | 19.7 | 19.8 | 19.8 | 19.8 | 19.9 | **407** | 726 | 236 | 384 | 532 | 680 | 828 | 976 |
| ***4A*** | ***Coinsurance – community care*** | **19.2** | 18.7 | 19.6 | 19.5 | 19.3 | 19.2 | 19.0 | 18.9 | **-269** | 151 | -533 | -378 | -222 | -67 | 88 | 244 |
| ***4B*** | ***Coinsurance – budget*** | **18.0** | 16.3 | 19.2 | 18.6 | 18.1 | 17.5 | 16.9 | 16.4 | **-278** | 133 | -538 | -388 | -237 | -86 | 64 | 215 |
|  | **Package** | **Interventions included in the benefits package** | | | | | | | | **Annual cases treated through the NHIS (millions)** | | | | | | | |
|  |  | **Base case**  **(14% / 22%)** | **Base case * 2 (29% / 43%)** | **5% / 10%** | **10% / 20%** | **15% / 30%** | **20% / 40%** | **25% / 50%** | **30% / 60%** | **Base case**  **(14% / 22%)** | **Base case * 2**  **(29% / 43%)** | **5% / 10%** | **10% / 20%** | **15% / 30%** | **20% / 40%** | **25% / 50%** | **30% / 60%** |
| ***1*** | ***Best buys*** | **39** | 39 | 39 | 39 | 39 | 39 | 39 | 39 | **47.7** | 47.7 | 47.7 | 47.7 | 47.7 | 47.7 | 47.7 | 47.7 |
| ***2*** | ***Status quo*** | **47** | 47 | 47 | 47 | 47 | 47 | 47 | 47 | **30.7** | 30.7 | 30.7 | 30.7 | 30.7 | 30.7 | 30.7 | 30.7 |
| ***3*** | ***Primary care*** | **40** | 40 | 40 | 40 | 40 | 40 | 40 | 40 | **49.0** | 49.0 | 49.0 | 49.0 | 49.0 | 49.0 | 49.0 | 49.0 |
| ***4A*** | ***Coinsurance – community care*** | **44** | 43 | 44 | 44 | 44 | 44 | 44 | 44 | **50.8** | 50.8 | 50.8 | 50.8 | 50.8 | 50.8 | 50.8 | 50.8 |
| ***4B*** | ***Coinsurance – budget*** | **46** | 46 | 46 | 46 | 46 | 46 | 46 | 46 | **50.5** | 50.5 | 50.5 | 50.5 | 50.5 | 50.5 | 50.5 | 50.5 |

**x% / y% headings explained: reduction of demand under 50% coinsurance on the left (x%) / reduction of demand under 100% coinsurance on the right (y%)

**Varying the budget:**

|  | **Package** | **Total annual NHB (millions DALYs avoided)** | | | **Net physician availability (FTE)** | | | **Interventions included in the benefits package** | | | **Annual cases treated through the NHIS (millions)** | | |
| --- | --- | --- | --- | --- | --- | --- | --- | --- | --- | --- | --- | --- | --- |
|  |  | **Base case** | **Reduced budget** | **Increased budget** | **Base case** | **Reduced budget** | **Increased budget** | **Base case** | **Reduced budget** | **Increased budget** | **Base case** | **Reduced budget** | **Increased budget** |
| ***1*** | ***Best buys*** | **20.1** | 19.9 | 20.1 | **-478** | -432 | -500 | **39** | 20 | 40 | **47.7** | 26.2 | 47.7 |
| ***2*** | ***Status quo*** | **18.2** | 18.2 | 18.2 | **-387** | -319 | -390 | **47** | 43 | 47 | **30.7** | 29.5 | 31.1 |
| ***3*** | ***Primary care*** | **19.7** | 19.8 | 19.7 | **407** | 443 | 346 | **40** | 38 | 41 | **49.0** | 47.9 | 50.1 |
| ***4A*** | ***Coinsurance – community care*** | **19.2** | 19.2 | 19.2 | **-269** | -186 | -285 | **44** | 37 | 46 | **50.8** | 48.8 | 51.1 |
| ***4B*** | ***Coinsurance – budget*** | **18.0** | 18.0 | 18.0 | **-278** | -252 | -291 | **46** | 43 | 47 | **50.5** | 49.6 | 51.2 |

**Assuming all HR costs are borne through the NHIS (as opposed to through the central government):**

|  | **Package** | **Total annual NHB (millions DALYs avoided)** | | **Net physician availability (FTE)** | | **Interventions included in the benefits package** | | **Annual cases treated through the NHIS (millions)** | |
| --- | --- | --- | --- | --- | --- | --- | --- | --- | --- |
|  |  | **Base case** | **All HR included** | **Base case** | **All HR included** | **Base case** | **All HR included** | **Base case** | **All HR included** |
| ***1*** | ***Best buys*** | **20.1** | 20.0 | **-478** | -464 | **39** | 31 | **47.7** | 37.3 |
| ***2*** | ***Status quo*** | **18.2** | 18.2 | **-387** | -363 | **47** | 46 | **30.7** | 30.2 |
| ***3*** | ***Primary care*** | **19.7** | 19.7 | **407** | 416 | **40** | 38 | **49.0** | 48.0 |
| ***4A*** | ***Coinsurance – community care*** | **19.2** | 19.1 | **-269** | -248 | **44** | 39 | **50.8** | 49.2 |
| ***4B*** | ***Coinsurance – budget*** | **18.0** | 18.0 | **-278** | -223 | **46** | 44 | **50.5** | 49.4 |

Cost-effectiveness threshold

Estimated total NHB decreases (increases) for all packages when the lowest (highest) estimate for the cost-effectiveness threshold is used. Varying the cost-effectiveness threshold had limited effect on the other three outcome measures.

Demand elasticity

Differences in total NHB between the packages become more marked as demand elasticity increases (i.e. estimated total NHB for packages *Best buys* and *Primary care* becomes higher, while estimated total NHB for the remaining packages becomes lower). Nonetheless, the relative ordering of the packages is unchanged. The higher the demand elasticity, the higher the net physician availability for all packages. This is explained by the reduced demand for uninsured care under higher demand elasticity, which means that less physicians are needed to meet total healthcare demand. Varying the demand elasticity has limited impact on the package-specific outcomes (‘number of interventions included’ and ‘cases treated through the NHIS’).

NHIS budget

While the interventions included and the cases treated decrease (increase) for all packages when a smaller (larger) budget is assumed, there is little change in the NHB outcome. The insensitivity of total NHB to a decrease in the budget may mean that the highly cost-effective interventions that bring large (net) population health can still be provided under a reduced budget. The insensitivity of total NHB to an increased budget, on the other hand, may mean that the additional interventions that can be added under a larger budget bring relatively small additional health benefits. However, since opportunity cost of healthcare spending (i.e. the appropriate cost-effectiveness threshold) likely changes as the budget for the benefits package changes, the NHB outcomes estimated using the original estimate of the cost-effectiveness threshold may be biased.

HR costs

When all HR costs are assumed to be paid through NHIS funding (as opposed to only HR costs for workers in private facilities), the number of interventions included in the package and number of cases treated decreases for all packages, while the net physician availability increases. Nonetheless, the relative ordering is unaffected.
